# Milk Powder Formulations with Varying Casein to Whey Ratios and Calcium Addition: Physico-Chemical and Structural Properties and the Effect of Low-Frequency Ultrasound

**DOI:** 10.3390/foods14040685

**Published:** 2025-02-17

**Authors:** Yuanyuan Zhao, Tuyen Truong, Jayani Chandrapala

**Affiliations:** 1 School of Science, STEM College, RMIT University, Bundoora, Melbourne, VIC 3083, Australia; yuanyuan.zhao@rmit.edu.au (Y.Z.); tuyen.truong@rmit.edu.vn (T.T.); 2 School of Science, Engineering and Technology, RMIT University, Ho Chi Minh City 700000, Vietnam

**Keywords:** ultrasound, whey protein, lactose, caseins, calcium, interactions, milk powder

## Abstract

This study examined the effect of low-frequency ultrasound (20 kHz, 1 and 5 min) on the physiochemical and structural properties of milk powder formulations with varying casein to whey ratios (0:100, 60:40, and 50:50) and calcium addition (30 mM). The ultrasound treatment led to changes in particle size, with an initial increase in aggregation followed by fragmentation. Calcium addition resulted in looser packing, as evidenced by a decrease in both bulk and tapped densities. DSC analysis indicated that calcium addition stabilized the protein–lactose matrix by increasing the glass transition temperature and reducing the number of thermal events. FTIR analysis revealed structural changes in proteins, with a decrease in β-sheet and β-turn and an increase in α-helix structures. These findings suggest that calcium plays a crucial role in reinforcing the structural integrity of the protein–lactose matrix, while ultrasound-induced mechanical forces lead to dynamic changes in particle size and protein conformation.

## 1. Introduction

Dairy powders are complex systems with varying proportions of caseins, whey proteins, lactose, fat, and minerals, influenced by the concentration process [1]. Lactose, proteins, and minerals are water-soluble, and their interactions with water and each other determine structure–property relationships [2]. The main milk proteins, casein and whey, have distinct properties where caseins are valued for their emulsifying, thermostability, and coagulation properties, making them essential in cheese production and other food applications [3]. Whey proteins are noted for their solubility, gelling, emulsifying, and foaming properties, which are advantageous in products like beverages, bakery items, and nutritional supplements [4,5]. Lactose, a disaccharide sugar, enhances sweetness and texture in products like sweetened condensed milk and infant formulas [6]. Calcium-fortified milk powders are developed for bone health, but careful management is needed to prevent issues such as phase separation, chalkiness, protein precipitation, and pH reduction when adding calcium salts [7,8,9,10,11,12,13].

Milk proteins and their interactions with other components significantly affect the characteristics and functional properties of dairy powders. Protein–calcium interactions influence milk’s heat stability [14,15]. During spray and freeze drying, lactose–protein interactions stabilize protein structures and prevent lactose crystallization [2,16,17,18,19,20]. Processing conditions such as pH, temperature, and drying methods further affect these interactions [21,22,23]. Additionally, lactose crystallization is influenced by mineral composition, and dairy products are also prone to the Maillard reaction due to their lactose and lysine content [24,25,26]. Research in dairy science has traditionally focused on two-way interactions among milk proteins, lactose, and minerals, with limited studies on their combined effects. In our previous study, we investigated these intricate interactions in liquid milk protein–lactose systems with varying casein-to-whey protein ratios (0:100, 50:50, 60:40, and 80:20), along with different concentrations of calcium chloride (CaCl_2_) (0–30 mM) [27]. We found that calcium concentration and pH significantly (*p* < 0.05) affected the physico-chemical and structural properties of the milk protein–lactose–calcium systems. Specifically, CaCl_2_ enhanced micellar stability in casein-rich solutions by promoting denser packing. Further investigation is needed to understand how these complex interactions between proteins, lactose, and minerals are affected when the liquid milk solution is converted to a dried form via spray drying, a common dairy processing technique. This knowledge will help dairy scientists formulate dairy powders with improved functionality.

Ultrasound is an emerging non-thermal process used in dairy for various advantages, including influencing milk proteins and their interactions with other components [28,29,30,31]. For instance, it has improved the crystallization of lactose by reducing crystallization induction times and increasing the rate of nucleation [32,33]. In our previous study, FTIR analyses showed that ultrasound promoted secondary structural changes in milk proteins and facilitated the formation of lactose–protein and calcium–lactose complexes. These interactions were dominated by intermolecular and intramolecular forces through hydrophobic and covalent bonding [27]. However, the effects of ultrasound technology on solid-state interactions among proteins, lactose, and minerals remain unexplored. It is still unclear whether ultrasound induces interactions or disrupts aggregates in such a three-component system, leaving a gap in fundamental understanding. Thus, this research aims to fundamentally understand the effects of low-frequency ultrasound (20 kHz, at 40% amplitude for 0, 1, and 5 min) on the interactions between proteins with varying casein to whey protein ratios, lactose, and minerals. It also seeks to examine how these interactions influence the physiochemical, thermal, and structural properties of milk protein–lactose powders with/without calcium addition. The findings of this study are highly relevant to the dairy industry, offering insights into developing dairy powders with improved functionalities and innovations in product formulations across applications like infant formula, sports nutrition, and functional foods.

## 2. Materials and Methods

### 2.1. Materials

Bovine milk protein isolate (MPI) (~85.5% protein, which is made up of 67.5% casein and 16.2% whey protein) and whey protein isolate (WPI) (88.8% whey protein) powders were purchased from Fonterra Co-operative Co., Ltd. (Richmond, VIC, Australia). Commercial food-grade α-lactose monohydrate powder with 99.6% purity and calcium chloride dihydrate powder (Molecular Weight: 147.01) were obtained from Sigma-Aldrich Pty. Ltd. (Castle Hill, NSW, Australia). Ultra-pure (Milli-Q) water was used as the liquid dispersant for all solution preparations.

### 2.2. Preparation of Reconstituted Concentrates

Three different reconstituted concentrates with casein (CN) to whey protein (WP) ratios of 60:40, 50:50, and 0:100, each with a total solid content of 20% *w/w*, were prepared following the method described in our previous study [27]. These samples are referred to as 64M (60:40), 55M (50:50), and WP-Control (0:100), respectively, throughout the following text. Lactose powder was dissolved in Milli-Q water at 22 ± 3 °C until achieving a clear solution containing 40 ± 0.5% (*w/w*) lactose. Calcium chloride solutions were formulated by dissolving a precise quantity of calcium chloride dihydrate powder in Milli-Q water at room temperature until complete dissolution occurred. The milk protein solutions were then mixed with the lactose and calcium solutions to attain final concentrations of 5% lactose and 30 mM calcium, and the mixtures were thoroughly blended using a magnetic stirrer at room temperature for 30 min to ensure homogeneity. The concentrates were stored at 4 °C overnight to promote hydration. The rehydrated solutions were subsequently subjected to ultrasonication and spray drying (as described below) within 12 h to ensure consistency in processing conditions.

### 2.3. Low-Frequency Ultrasound Treatment

The reconstituted concentrates (500 mL) were subjected to low-frequency ultrasound (20 kHz) treatment at 40% amplitude for 0, 1, and 5 min, as described previously [27]. During sonication, the samples were kept in an ice bath to maintain the temperature below 10 °C. The temperature was checked after each treatment using a digital thermometer (Brannan, Australian Scientific Pty Ltd., Kotara, NSW, Australia).

### 2.4. Laboratory Scale Spray-Drying of Milk Powders

Spray drying was performed on both sonicated and unsonicated concentrates using a laboratory spray dryer B290 (Buchi Labortechnik AG, Flawil, Switzerland), with inlet and outlet temperatures set to 180 °C and 80 °C, respectively. The liquid feed flow, nozzle orifice size, and airflow rate were 16 mL/min, 0.7 mm, and 35 m^3^/h. To ensure temperature consistency and minimize the impact of initial temperature on spray-drying results, all samples were warmed in a hot water bath to approximately 20 ± 1.0 °C before drying. The resulting powders were collected in airtight containers and stored at room temperature in a desiccator to prevent moisture absorption. The powders were stored for no more than 2 h before being subjected to further analyses.

### 2.5. Particle Size Distribution

Mastersizer 3000 laser diffraction particle size analyzer (Malvern Instruments Ltd., Worcestershire, UK) equipped with a powder sampler (Aero M, Malvern Instruments Ltd.) was used to evaluate particle size distributions of the concentrates and powders. Based on the Mie theory, the optical model was used on the measurement with the following conditions: refractive index, 1.56; air pressure, 2 bar and feed rate, 25% [34,35]. The results of particle size analysis were expressed as follows: D[4,3] (volume mean diameter); D[3,2] (surface area mean diameter); and d(0.1), d(0.5), and d(0.9) (10%, 50%, and 90% of particles in the sample were below these diameters, respectively).

### 2.6. Bulk Density

A bulk density assessment was performed as follows: 5 g of powder was accurately weighed using an analytical balance and transferred into a 10 mL graduated glass cylinder. The initial volume of the powder (Vbulk) was recorded. The cylinder was then tapped 100 times until no further reduction in volume was observed, and the final tapped volume (Vtapped) was noted.

Bulk density (g/mL) was calculated as:

Bulk density = m/Vbulk

Tapped density (g/mL) was calculated as:

Tapped density = m/Vtapped

where m represents the mass of the powder (5 g).

### 2.7. Color Measurements

To evaluate the color parameters of powder samples, a Minolta Chroma Meter CR-400 (Minolta, Osaka, Japan) was used. The assessments were carried out at room temperature (25 °C). CIELAB color space was used, determining the parameters L* (lightness or whiteness, black = 0, and white = 100), a* (redness > 0, greenness < 0), and b* (yellowness > 0, blue < 0) for each sample.

### 2.8. Solubility Test

The powder samples were dissolved in water to a final dry weight of 10% (*w/w*). The solutions were centrifuged at 1000× *g* for 10 min at 20 °C (Sigma, 3-30KS, Osterode am Harz, Germany). A sample of the supernatant was placed in a pre-weighed moisture dish and weighed. The dish was dried in an oven at 105 °C overnight, then cooled in a desiccator to avoid condensation effects [36], and then reweighed. The solubility of the powder samples was calculated as the total solids content of the supernatant, expressed as a percentage of the total solids content of the initial solution [36,37].

### 2.9. Moisture Content and Water Activity

The moisture content of the samples was measured by a moisture analyzer (Ohaus Halogen MB Series; MB 45, Greifensee, Switzerland) at 105 °C. The moisture content obtained was expressed as a percentage on a wet-weight basis. The water activity (aw) of the powder samples was measured at 25 °C using an AquaLab Water Activity Meter Series 3TE (Decagon Devices, Inc., Pullman, WA, USA) with internal temperature control. For accurate water activity measurement, enough powder was added to fill half of the sample dish, following the manufacturer’s guidelines.

### 2.10. Thermal Analysis

Thermal characteristics of spray-dried powders were measured by differential scanning calorimetry (DSC, Q2000, Mettler Toledo, Schwerzenbach, Switzerland). Aluminum sample pans, which were hermetically sealable, were used in all measurements with an empty pan as the reference. Approximately 5 ±  0.05 mg of a powder sample was transferred into the aluminum pan, which was then hermetically sealed and reweighed. The powders were scanned from 30 to 250 °C at a heating rate of 5 °C min^−1^ to capture the full range of thermal events.

### 2.11. FTIR Measurement

A Perkin Elmer Frontier FTIR spectrometer (Perkin Elmer, Waltham, MA, USA) was used to collect the Fourier-transform infrared spectra (FTIR) in the 4000–400 cm^−1^ range. An average of 32 scans was recorded at a resolution of 4 cm^−1^. Special attention was paid to original spectra in the region between 1200 and 850 cm^−1^ and the second derivative of region 1700–1600 cm^−1^ (Amide II) for evaluating structural changes in carbohydrates and proteins, respectively [38]. The second-derivative spectra were calculated using the Savitzky-Golay derivative algorithm [39]. The relative contents of the secondary structures of protein were calculated from the areas of the individual bands of the Gauss curve-fitting result [38].

### 2.12. Statistical Analysis

The entire experiment was conducted in triplicate, with each measurement performed at least in duplicate. The results are presented as the average ± standard deviation. Data were analyzed using the General Linear Model (GLM) in Minitab statistical software Version 21.1.0 (Minitab, LLC, State College, PA, USA) to examine both main effects and interaction effects. A one-way ANOVA was used to compare the means of the samples with three replicates, followed by a post hoc Tukey comparison test to determine significant differences. Statistical significance was set at a probability level of 5% (*p* < 0.05).

## 3. Results and Discussion

### 3.1. Physico-Chemical and Functional Characterisations

This study involved the production of 13 milk powder samples to examine the effects of varying casein-to-whey ratios, ultrasonication durations, and calcium chloride additions, as illustrated for the visual appearance in Appendix A.

**Figure 1:**
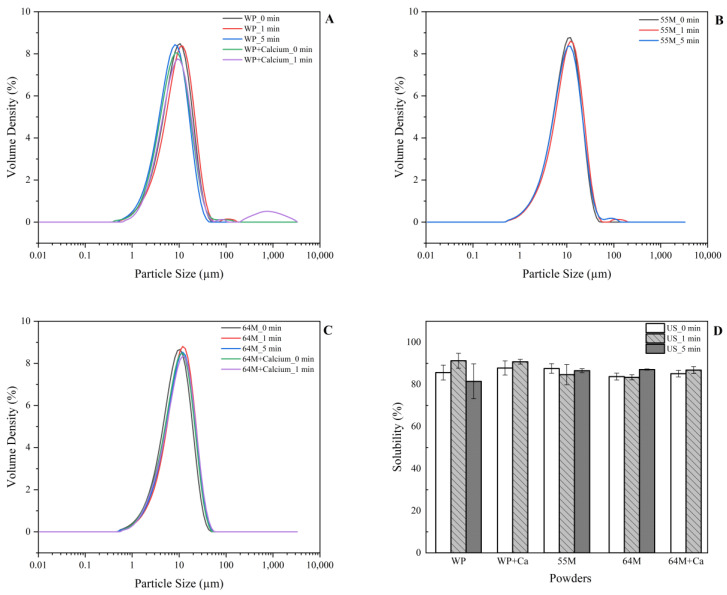
Particle size distribution (**A**–**C**) and solubility (**D**) of milk powder samples containing varied casein to whey protein rations (0:100, 60:40, and 50:50, denoted as WP, 64M, and 55M) and CaCl_2_ level (0 and 30 mM) as a function of sonication time (0, 1, and 5 min).

Particle size: The particle size values (D[4,3], D[3,2], and d(50)) are summarised in Appendix B, while the particle size distribution (D[4,3]) and solubility of all studied dairy powders are illustrated in Figure 1. All unsonicated samples without calcium addition (WP, 55M, and 64M) exhibited a uniform particle size distribution with a sharp peak around 10 µm. However, after 1 min of sonication, particle aggregation was induced in WP (Figure 1A) and 55M (Figure 1B) samples, resulting in a bimodal distribution. This aggregation is likely due to the formation of protein aggregates following sonication, consistent with previous studies [40,41]. With the extended sonication to 5 min, the bimodal size distribution of the WP sample changed to monomodal distribution, with a leftward shift in the main peak indicating particle fragmentation. It is likely that the change in particle size distribution is attributed to ultrasound (US)-induced cavitation shear forces, which are more effective in breaking up large multimeric protein–protein aggregates than individual monomeric proteins [42].

However, the casein-rich samples (64M) showed no significant change in particle size distribution, even after sonication. This observation suggests that the higher casein content may provide greater structural resistance to the effects of sonication, such as aggregation or fragmentation, compared to whey-dominant formulations. The inherent stability of casein micelles, which are known to form stronger networks due to calcium-mediated cross-linking, may explain why casein-rich powders are less susceptible to changes under sonication. This resistance is consistent with observations that higher casein content can lead to more stable particle structures both in liquid and dried dairy systems.

Despite these changes in particle size distribution, statistical analysis indicated that there was no statistically significant difference in D[4,3] values (Table 1). This suggests that while aggregation occurred, it was not substantial enough to result in a statistically meaningful change in overall particle size. Further statistical analyses on other particle size values, D[3,2] and d(50) showed that casein to whey ratios (*p* < 0.01) had a significant impact on particle size for both D[3,2] and d(50), further emphasizing the role of protein composition in determining particle under sonication. Interaction effects were observed between ultrasonication, protein composition, and calcium level (Table 1) regarding D[3,2] values, highlighting the importance of optimizing protein ratios and calcium concentrations to control particle size characteristics.

Rehydration characteristic. Figure 1D presents the powder solubilities of all studied samples in the range of 80–90%. This indicates the product’s ability to dissolve efficiently in water (smooth, lump-free solution). High solubility ensures better reconstitution in various applications, from infant formula to dairy beverages, enhancing both the texture and nutritional delivery. In this study, no significant main effects or interaction effects were found regarding milk powder solubility when considering the factors of milk mixture composition, calcium level, and ultrasound duration (Table 1). This indicates that variations in the casein-to-whey protein ratios, calcium concentrations, and ultrasound treatment times did not independently or collectively influence the solubility of the milk powder. However, it is important to note that these results are based on freshly made samples. Storage conditions may impact solubility over time, potentially altering powder structure or composition and, consequently, its dissolution behavior.

In this study, Tukey’s test showed a significant difference in solubility between the WP sample, which underwent 1 min (90.2%) and 5 min ultrasonication (81.4%) (*p* < 0.05). The WP-Control powder sample exhibited aggregate formation after 1 min of sonication, yet solubility increased. However, after 5 min of sonication, the aggregates were broken down, and solubility decreased significantly. An increase in particle size alongside improved solubility can occur due to several factors related to the structural and compositional changes within the particles. For instance, larger particles may possess a more porous structure, allowing water to penetrate more easily and facilitating faster dissolution. Additionally, changes in surface composition—such as the distribution of hydrophilic compounds—can enhance water interaction [43,44], even if the particle size is larger. This behavior may be attributed to the cavitation effects caused by sonication, which increase the local temperature and pressure around collapsing bubbles, leading to protein unfolding [45]. This unfolding initially results in changes in protein conformation, with hydrophilic amino acid residues becoming reoriented toward water, thereby enhancing solubility [46]. Similarly, Fredenberg et al. [47] stated that the high pressure generated by acoustic cavitation induces an open protein state where water easily permeates. This, in turn, replaces the intramolecular hydrogen bonds with water–protein bonds, and the strong water–protein hydrogen bonds lead to an intact secondary protein structure. However, with prolonged sonication, additional processes likely come into play. Extended sonication can expose hydrophobic regions or lead to partial denaturation, which reduces solubility. This counteracting effect may explain why ultrasonication alone did not significantly influence solubility across the samples (*p* > 0.05, Table 1) in this study.

The direct addition of calcium without sonication resulted in only minor changes to the particle size distribution of the WP-Control sample. However, after 1 min of sonication in the presence of calcium, the WP+Ca samples exhibited a trimodal distribution with peaks between 500 µm and 2000 µm, indicating particle aggregation. This suggests that calcium in combination with sonication enhances the likelihood of aggregation in WP samples by promoting protein cross-linking during sonication. Despite this aggregation, the solubility of the WP+Ca samples remained relatively stable, as shown in Figure 1C, with no significant changes in solubility detected (*p* > 0.05). This indicates that changes in particle size distribution do not necessarily correlate with solubility. Statistical analysis further confirms that neither calcium addition nor sonication significantly affected the solubility of WP samples (*p* > 0.05).

Although there was no statistical difference, regardless of ultrasound treatment, the solubility of 64M samples containing 30 mM calcium tended to have higher solubility (86.8–87.8%), except WP-Control 30 mM with 1 min sonication. This suggests that the presence of casein, which forms stable micelles combined with calcium, helps to preserve the structural integrity of the particles, preventing significant aggregation and solubility loss. This behavior is consistent with the findings of Chandrapala et al. [48], who noted that while sonication reduces the size of whey protein aggregates, it does not affect casein micelles, their composition, or mineral balance in fresh skim milk. In comparison, the solubility of the 55M sample showed different trends: short-term sonication led to a decrease in solubility, whereas prolonged sonication resulted in an increase, although this change was not statistically significant (*p* > 0.05). In contrast, the 64M sample showed relatively constant solubility after 1 min of sonication, with a slight increase after 5 min. This could be due to the higher proportion of casein, which forms stable micelles that are less responsive to the shearing forces of sonication.

Coloration: Table 1 shows that all color parameters (L*, a*, b*) of dairy powder samples were significantly impacted (*p* > 0.05) by the main effects of varying casein to whey protein ratios and their interaction with sonication duration. The a* values appear to be sensitive to all variables with the main effects of calcium level and sonication duration but not the b* values.

Minimal changes in lightness (L*) were observed for all samples, irrespective of sonication, protein ratio, or addition of calcium (Table 2). However, the changes in b*, which denote the yellowness of the samples, were influenced by sonication, protein ratio, and the addition of calcium. For WP samples, sonication led to a slight decrease in yellowness, but this decrease was not statistically significant (*p* > 0.05). This decrease may be due to the disruption of protein structures during sonication, which could reduce the availability of free amino groups that participate in the Maillard reaction. The statistical analysis confirmed that protein ratio (milk mixture) and calcium level significantly influenced b* (*p* < 0.001 and *p* < 0.05, respectively, Table 1), suggesting that the composition and calcium presence played more critical roles in color changes than sonication alone.

In contrast, the 55M samples showed a notable increase in b* after 1 min of sonication (*p* < 0.05), likely due to the initial enhancement of the Maillard reaction as sonication-induced cavitation increases temperature locally, accelerating the reaction [49]. However, with prolonged sonication, the b* value decreased significantly (*p* < 0.05), resulting in the lowest yellowness compared to the control 55M without sonication. This reduction in yellowness may be attributed to the breakdown of larger Maillard reaction products into smaller, less pigmented molecules or the disaggregation of protein aggregates, reducing the Maillard reaction’s impact. Similarly, the 64M sample exhibited an increase in b* after 1 min of sonication (*p* < 0.05), potentially due to an enhanced Maillard reaction under the influence of sonication. Unlike 55M and WP, 64M showed a further significant increase in b* with prolonged sonication (*p* < 0.05). This suggests that in the 64M samples, the Maillard reaction continued to progress or that other structural changes in the protein–lactose matrix facilitated further pigment formation. This finding aligns with previous research by Cardoso et al. [50], which reported that α-lactalbumin was slightly more reactive than β-lactoglobulin for all protein–saccharide combinations and that β-casein possessed, on average, 45% lower reactivity than the whey proteins. Although casein is less matrix, its presence along with more reactive whey proteins, could contribute to this increase in yellowness in the 64M sample.

The addition of calcium to the WP sample resulted in a decrease in the b* value from 3.93 to 3.87 at 0 min of sonication and a more pronounced decrease to 3.01 after 1 min of sonication (*p* < 0.05). The reduction in yellowness with calcium addition suggests that calcium may stabilize the protein structure, reducing the potential for Maillard reactions, especially in the less reactive whey protein-rich sample. For the 60:40 sample with added calcium, the b* value increased from 3.82 to 4.23 at 0 min of sonication (*p* < 0.05), followed by an increase to 4.57 after 1 min of sonication (*p* < 0.05). Unlike in the WP sample, the presence of calcium in the 64M sample may have facilitated a slight increase in Maillard reaction activity, potentially due to calcium’s interaction with casein, which could alter the protein matrix and enhance browning despite casein’s lower reactivity.

Bulk densities. The bulk density of the powders ranged from 0.25 to 0.33 g/cm^3^, while the tapped density ranged from 0.35 to 0.49 g/cm^3^, irrespective of the addition of calcium. The varying casein-to-whey ratios are found to impact the powder densities (*p* < 0.05). Sonication slightly altered the densities of the powder by 6–10% for all samples (*p* > 0.05). The additional of calcium, especially when combined with sonication, had a more pronounced impact, reflected by their significant interaction effects (Table 1), particularly in the 64M sample. In WP+30 mM Calcium, sonication resulted in a slight decrease in both bulk (from 0.27 to 0.25 g/cm^3^) and tapped densities (from 0.38 to 0.35 g/cm^3^), but this was not statistically significant (*p* > 0.05). However, the 64M Ca-added sample with 1 min of sonication showed a significant decrease in both tapped and bulk density compared to its non-Ca-added sample (*p* < 0.05). This specific behavior can be attributed to the higher casein content in the 64M sample, which interacts more effectively with calcium. Casein proteins are known to form strong complexes with calcium ions, leading to changes in the micellar structure. When sonication is applied to the 64M Ca-added sample, it likely causes the casein micelles to partially disintegrate or reorganize, increasing the volume of interstitial spaces between particles. This reorganization could lead to more air inclusion during the spray drying process, particularly as moisture evaporates, resulting in a significant decrease in both bulk and tapped densities [51].

Moisture contents: The moisture content of the WP powders did not change significantly with sonication. In contrast, the 55M and 64M samples experienced more pronounced changes in moisture content. After 5 min of sonication, the moisture content of the 55M sample significantly decreased from 3.54% to 3.28% (*p* < 0.05), indicating greater moisture removal during spray drying. Similarly, the 64M sample showed a notable decrease in moisture content from 3.56% at 0 min to 3.21% after 1 min of sonication and further decreased to 3.20% after 5 min of sonication (*p* < 0.05). The more significant moisture reduction in the 55M and 64M samples compared to WP can be attributed to their higher casein content. Casein proteins, particularly in the form of micelles, have a more open and porous structure compared to the tightly folded globular structure of whey proteins [52]. This porous nature allows caseins to interact more extensively with water molecules, leading to higher initial moisture retention. However, during sonication, the cavitation may reduce the water-holding capacity of the casein molecules. As a result, more moisture is released from the casein-containing powders during the spray-drying process. Moreover, the interaction between casein and calcium, which is more prominent in the 64M samples, can further enhance this effect. Calcium can bridge casein micelles, leading to denser, less hydrated structures after sonication. This reduction in hydration makes it easier for moisture to be removed during spray drying, resulting in a lower final moisture content in the sonicated 55M and 64M powders.

The addition of calcium resulted in a significant increase in moisture content (*p* < 0.05), irrespective of the protein ratio, with both WP and 64M samples showing approximately a 33% increase for both sonicated and non-sonicated conditions. This increase in moisture content can be attributed to the hygroscopic nature of calcium salts. Calcium ions have a strong affinity for water molecules, which can lead to increased moisture retention in the protein–lactose matrix of the powders. Additionally, calcium can interact with proteins and lactose, forming complexes that alter the microstructure of the powder particles. These calcium–protein–lactose complexes can trap more water within the matrix, increasing the overall moisture content. This is particularly evident in systems containing casein (such as 64M), where calcium ions can bridge casein micelles, leading to denser structures that retain more moisture.

Water activity: Water activity also increased with the addition of calcium, irrespective of sonication and protein ratio. The rise in water activity can be explained by the fact that calcium ions, by binding water molecules more effectively, increase the amount of free or “unbound” water in the system. This binding effect, coupled with calcium-induced structural changes in the protein–lactose matrix, creates more sites for water interaction, further contributing to the increased water activity. The statistical analysis showed that the influence of calcium was significant (*p* = 0.008), while sonication duration and the interaction between calcium and sonication did not significantly affect water activity (*p* > 0.05). This effect is consistent across different protein ratios and is independent of sonication, as the presence of calcium uniformly influences the water-binding capacity of the powders.

### 3.2. Differential Scanning Calorimetry

**Figure 2:**
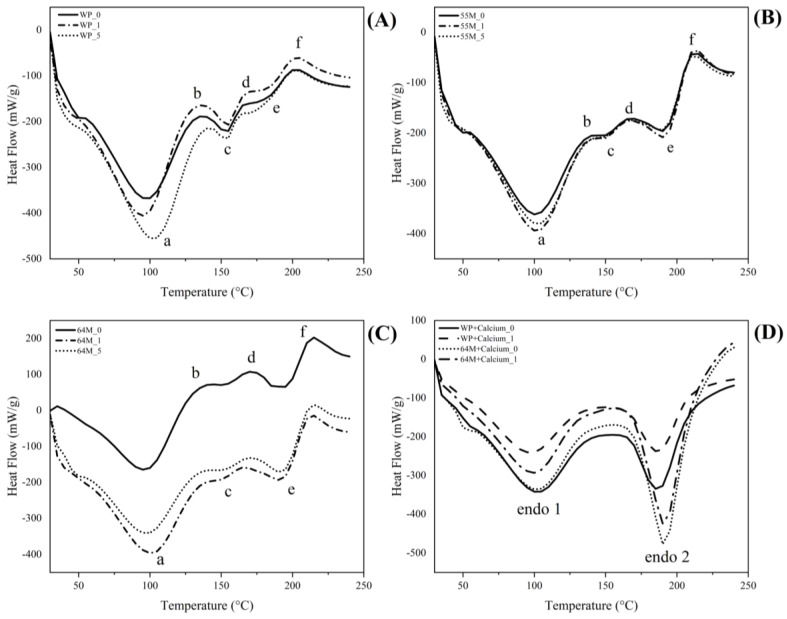
DSC curves of the milk powders under flowing N_2_ atmosphere at 5 K min^−1^. Main peaks (a–f) were indicated with lowercase letters for samples without calcium. The subfigures represent: (**A**) WP; (**B**) 55M; (**C**) 64M; and (**D**) sample with calcium addition.

Figure 2 shows the DSC curves obtained for the studied samples displaying multiple thermal events over the entire temperature range (30–250 °C). The first notable feature in the DSC curves is the glass transition temperature (Tg), observed as a step change in the heat flow, marking the transition from a glassy, rigid state to a more flexible, rubbery state [53,54]. In the unsonicated WP sample, a Tg is observed at approximately 60 °C, which is attributed to the amorphous lactose present in the sample. This transition reflects the temperature at which the amorphous lactose becomes more mobile and less rigid. Interestingly, as shown in Figure 2A, increasing sonication time results in a noticeable reduction in Tg, suggesting an increase in molecular mobility and further amorphization of the lactose. This change is associated with enhanced solubility, as amorphous structures generally dissolve more readily than crystalline forms. However, excessive amorphization may increase hygroscopicity, making the powder more prone to moisture absorption, which can negatively impact particle stability. The observed reduction in Tg aligns with structural changes induced by sonication, where the mechanical energy from sonication disrupts lactose crystals, leading to the formation of an amorphous matrix. While many studies, such as those by Dincer et al. [33], indicate that sonication often promotes lactose crystallization, the presence of proteins like whey protein in the current study appears to inhibit this crystallization process. Furthermore, Sánchez-García et al. [55] reported that while whey proteins can accelerate the rate of lactose crystallization, they also contribute to the maintenance of a higher level of amorphous lactose. This suggests that the interaction between whey proteins and lactose not only influences the crystallization process but may also stabilize amorphous regions within the matrix, ultimately affecting its functional properties. The 55M sample similarly showed a glass transition event around 60 °C, consistent with the WP sample. However, the transitions in the 55M are less pronounced, suggesting a less stable amorphous lactose matrix due to the presence of caseins.

Unlike the WP sample, the Tg in 55M shifted to higher temperatures with sonication. This shift to higher Tg could be due to the interactions between casein molecules and lactose, which may restrict molecular mobility, making it more resistant to the disruptions typically caused by sonication. The 64M sample exhibited an event around 60 °C, similar to the WP and 55M samples. However, this transition was broader and more spread out, suggesting a more heterogeneous amorphous state within the matrix. This heterogeneity could be due to the higher casein content in 64M, which might create a less uniform amorphous structure compared to WP and 55M. With sonication, the Tg in the 64M samples shifts to lower temperatures. This shift is more pronounced in the 64M samples than in WP but less so than in 55M. The more significant Tg shift in 55M, despite its higher whey protein content compared to 64M, suggests that the specific casein-to-whey protein ratio in 55M may promote different types of protein–lactose interactions. In 55M, the nearly equal ratio of casein to whey protein likely leads to a more dynamic and unstable matrix, resulting in greater structural changes upon sonication. This contrasts with 64M, where the higher casein content seems to interact differently with the whey protein and lactose, leading to a more stabilized structure. The DSC curves indicate that calcium addition stabilizes the amorphous lactose matrix, leading to higher glass transition temperatures. Specifically, for the WP sample, the Tg increased from 60 °C to around 65 °C with calcium addition. This stabilization likely occurs because calcium interacts with lactose and protein molecules, forming a more rigid and cohesive matrix that resists molecular mobility. Calcium ions can bridge lactose and protein molecules through ionic interactions, enhancing the structural integrity of the amorphous matrix and raising the Tg. However, sonication disrupts this stability, causing shifts to lower Tg values. For example, after 1 min of sonication, the Tg of the calcium-added WP sample decreased to around 58 °C. The energy from sonication introduces mechanical forces and localized heating (cavitation effects), which disrupt these calcium-induced interactions, leading to increased molecular mobility and a subsequent decrease in Tg.

The powder samples without calcium showed three endothermic and three exothermic peaks across all ratios, irrespective of sonication. The first prominent endothermic peak was observed around 100–105 °C which can be attributed to the denaturation of specific milk proteins, including peptides, polypeptides, and globulin peptides, depending on the exact location [56,57], Szulc et al. [58] reported an endothermic transition at 104.2 °C associated with β-lactoglobulin denaturation in industrial-scale milk powders [59].With sonication, this peak shifted to a higher temperature in the WP and 64M samples, moving from approximately 100–105 °C to around 107–110 °C. At the same time, no change was observed for 50M. This shift to higher temperatures in WP and 64M suggests increased thermal stability of the proteins, possibly due to changes in protein structure or aggregation states induced by sonication. The increased stability could result from protein-protein interactions that form during sonication, which require more energy (i.e., higher temperature) to break apart. When examining the enthalpy associated with this peak (the area under the curve), a decrease in enthalpy was observed with sonication for WP and 64M samples, indicating that less energy was required for the denaturation process. This reduction in enthalpy could suggest partial unfolding or changes in the aggregation state of the proteins, making them less resistant to thermal denaturation. In contrast, the enthalpy for the 55M sample remained relatively unchanged, suggesting that the structural changes induced by sonication were less significant in this ratio. This difference in behavior between the samples may be attributed to the varying interactions between casein and whey proteins in each formulation, with certain ratios promoting more stable protein complexes that are less affected by sonication.

The other two endothermic peaks were noted around 155 and 190 °C, corresponding to water evaporation from α-lactose monohydrate and the onset of lactose melting, respectively [58,60,61,62]. Interestingly, the 155 °C range endothermic peak showed no change for both 55M and 64M, and also, the peaks are smaller, indicating only partial crystallinity samples irrespective of sonication. In contrast, WP samples showed a lower-end shift in temperature with an increase in sonication time (from 155 to 150 °C). This observation is in line with FTIR result (shown below). The lactose melting peak was much more prominent with 50M and 64M samples as compared to WP samples. However, the onset nor the enthalpy were significantly different as a function of sonication for both 50M and 64M samples. This suggests that while sonication might influence the degree of crystallinity or the thermal stability of lactose to some extent, the fundamental melting behavior of lactose remains consistent in these samples. Additionally, an exothermic peak at approximately 140 °C, denoted as “b” in the DSC curves, was observed across all samples (without calcium). This peak is likely associated with the early stages of the Maillard reaction, where reducing sugars like lactose begin reacting with amino groups in proteins, leading to initial chemical changes such as browning and flavor development. Unlike the 50M and 64M samples, which showed no change in this peak, the WP samples exhibited a shift towards higher temperatures with increasing sonication time. This suggests that the Maillard reaction in the WP samples is more sensitive to sonication, potentially due to the unique interaction dynamics between lactose and whey proteins in the absence of casein. Following this, the second exothermic peak around 175 °C, associated with the transition of amorphous lactose, was also observed, consistent with previous studies indicating the recrystallization of lactose in this temperature region [20,62,63]. This peak again showed no change for both 50M and 64M samples, although WP samples showed a shift towards higher temperatures as a function of sonication. In the WP samples, sonication likely induces structural changes in whey proteins, such as unfolding and aggregation, which could alter the local environment around the lactose molecules. These changes may hinder the mobility and interaction of lactose molecules, thereby requiring more energy (i.e., a higher temperature) for recrystallization to occur. Additionally, the absence of casein in the WP samples might mean there are fewer competing interactions, allowing the whey proteins to more directly influence the recrystallization process of lactose. The last exothermic peak was observed above 200 °C. The peak was a result of sample decomposition (lactose crystallization, Maillard reaction, and/or decomposition and denaturation of proteins or carbohydrates with the formation of new compounds). The onsets did not change as a function of sonication, indicating no effect on sample decomposition.

The presence of calcium simplified the DSC profiles, reducing the number of distinct thermal events (Figure 2D). Only two prominent endothermic peaks around 100–105 °C and 190 °C were observed. The first peak, labeled as “endo 1”, can be attributed primarily to moisture loss or water evaporation, consistent with the temperature range where bulkily bound water typically evaporates. The onset temperature for this peak shifted slightly higher with calcium addition, from approximately 100 °C in the WP sample without calcium to around 103 °C in the WP+Ca sample. This shift suggests that calcium may be stabilizing the moisture content within the matrix, making it slightly more resistant to evaporation. The enthalpy associated with this peak also increased with calcium addition, indicating that more energy is required for water loss in the presence of calcium, likely due to stronger interactions between water molecules and the calcium-protein–lactose matrix. The second peak, labeled as “endo 2”, was around 190 °C; this likely signifies the melting of crystalline lactose, reflecting the energy absorption required for the transition from a solid crystalline state to a liquid state. In the WP sample without calcium, this peak appeared at around 188 °C, but with calcium addition, the onset temperature slightly increased to approximately 191 °C. This shift to a higher temperature suggests that calcium enhances the thermal stability of crystalline lactose, possibly by strengthening the crystalline lattice or through interactions with lactose molecules that make the crystalline structure more resistant to melting. The enthalpy associated with this second peak also showed an increase with calcium addition, indicating that more energy is required for the melting transition, which reflects the increased stability of the crystalline lactose structure in the presence of calcium. Similarly, for the 64M samples, the addition of calcium led to comparable changes in the DSC profiles. The onset temperature for “endo 1” shifted from about 102 °C in the 64M sample without calcium to around 105 °C in the 64M+Ca sample, and the enthalpy of this peak also increased, indicating a more stable moisture environment within the matrix. The second endothermic peak at 190 °C also exhibited a higher onset temperature with calcium addition, shifting from around 189 °C to approximately 192 °C, with an accompanying increase in enthalpy. This indicates that the presence of calcium in the 64M sample similarly stabilizes the crystalline lactose, requiring more energy for the melting transition compared to the non-calcium sample.

When considering the effects of sonication on the WP and 64M samples with and without calcium, notable differences in both onset temperatures and enthalpy values were observed. For the WP+Ca sample, sonication caused a slight decrease in the onset temperature of both “endo 1” and “endo 2” peaks, with the “endo 1” peak moving from 103 °C to about 101 °C and the “endo 2” peak shifting from 191 °C to 189 °C after 5 min of sonication. The enthalpy for both peaks also decreased, suggesting that sonication disrupts the stabilizing effect of calcium, making the structure more susceptible to thermal transitions. In the 64M+Ca sample, sonication similarly reduced the onset temperatures of both peaks, though the changes were less pronounced than in the WP+Ca sample. The “endo 1” peak shifted from 105 °C to around 103 °C, and the “endo 2” peak moved from 192 °C to 190 °C with sonication. The enthalpy also decreased, but to a lesser extent than in the WP+Ca sample, indicating that the higher casein content in 64M may provide additional stabilization against the disruptive effects of sonication.

### 3.3. FTIR Analysis

**Figure 3:**
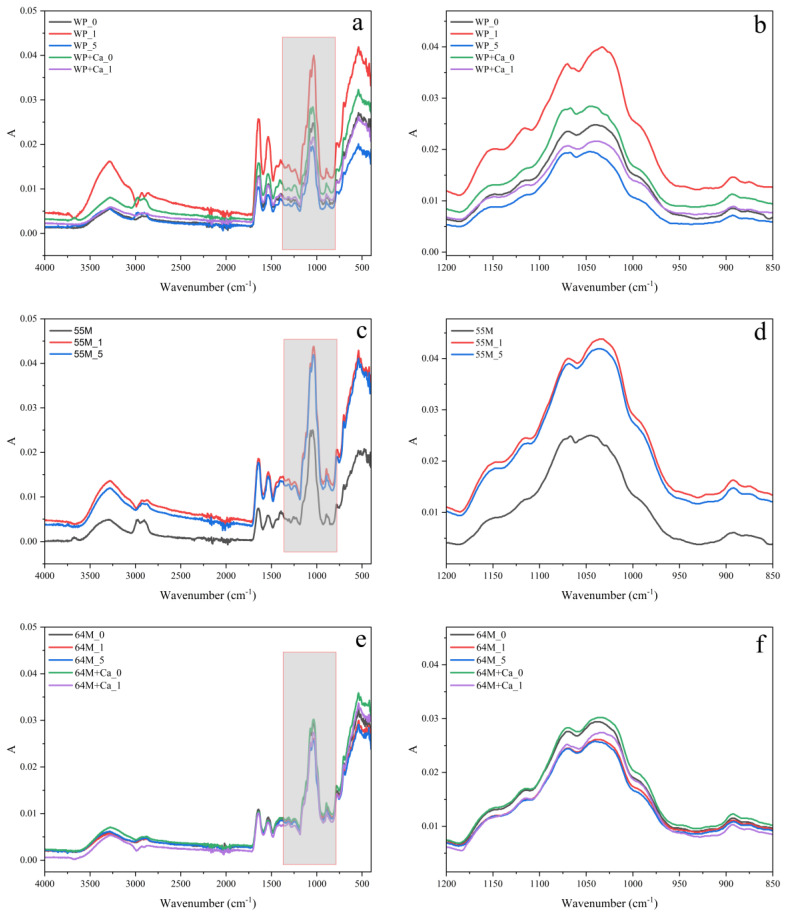
FTIR spectra of all studied powders: (**a**,**c**,**e**) in the range 4000–400 cm^−1^ and (**b**,**d**,**f**) in the range 1200–850 cm^−1^. Samples include WP, 55M, and 64M with variations of calcium addition and ultrasound treatment durations (0, 1, and 5 min).

The FTIR spectra presented in Figure 3 were registered in the classic range (4000–400 cm^−1^) and a specific sub-range, 1200–850 cm^−1^.

#### 3.3.1. The 1200–850 cm^−1^ Region

In the FTIR spectra, the region from 1200 to 850 cm^−1^ is associated with carbohydrate-related vibrations [64]. The presence of sharp, well-defined peaks in the FTIR spectra of the WP, 55M, and 64M samples before sonication strongly suggests that the lactose in these samples is predominantly in a crystalline state [60]. The WP sample has a greater number of peaks and more distinct sharp peaks compared to the 55M and 64M samples, indicating a highly crystalline lactose structure, which aligns with the DSC results discussed above. The 55M and 64M samples, with higher casein content, show similar but slightly different peak patterns compared to each other, reflecting variations in their crystalline lactose structure.

Sonication-induced a transition from a predominantly crystalline to a glassy (amorphous) state of lactose in WP, but retaining a significant crystalline component event after prolonged treatment. In contrast, the 55M samples experienced significant amorphization after sonication treatment. Sonication for 1 min resulted in notable structural modifications, with the appearance of new peaks at 1148, 1116, 1070, 1036, 922, and 893 cm^−1^, and shifts in existing peaks. After 5 min of sonication, the peaks shifted to 1148, 1113, 1069, 1038, 920, and 892 cm^−1^, showing substantial disruption of the crystalline structure and a higher degree of amorphization compared to WP. These peak shifts correspond to changes in molecular vibrations associated with lactose–protein interactions and hydrogen bonding, suggesting increased structural rearrangement in the system [40,60,64]. The 64M samples show intermediate changes, with higher casein content mitigating the disruptive effects of sonication compared to 55M. As evidenced by the new peaks at 1148, 1116, 1071, 1041, 919, and 894 cm^−1^ that appeared after 1 min of sonication. Prolonged sonication for 5 min resulted in peaks at 1149, 1114, 1071, 1041, and 892 cm^−1^, indicating increased amorphization while retaining some crystalline structure, similar to WP but less pronounced than in 55M. These findings highlight that the protein ratio significantly influences the impact of sonication on lactose, with casein presence leading to more substantial structural alterations. The observed shifts in FTIR peaks indicate increased molecular disorder, which can affect powder reconstitution properties. As previously discussed, a higher degree of amorphization generally enhances solubility and dispersion, as amorphous structures dissolve more readily. Studies have shown that ultrasound reduces the formation of amorphous lactose in the presence of whey proteins [55,65]. This reduction is due to sonocrystallization, which decreases the incorporation of β-lactose into the crystals, thereby improving the crystallinity of lactose [55,66]. This is possibly the reason why WP showed a minor change in lactose state along with the sonication time compared to others.

The addition of calcium to WP results in fewer and broader peaks with slight shifts, indicating a reduction in crystallinity or partial amorphization. Sonication for 1 min in the presence of calcium results in further shift (e.g., 1151 to 1150 cm^−1^ and 1116 to 1115 cm^−1^) and the appearance of new peaks (e.g., 930 and 924 cm^−1^). The combination of calcium and sonication leads to more pronounced structural changes, reducing the crystalline structure of lactose and increasing in amorphous content. The 64M samples show similar trends in terms of peak shifts and broadening with calcium addition and sonication. However, the specific positions and the degree of change may differ due to the different protein ratios and interactions with lactose.

#### 3.3.2. The 1700–1600 cm^−1^ Region

**Figure 4:**
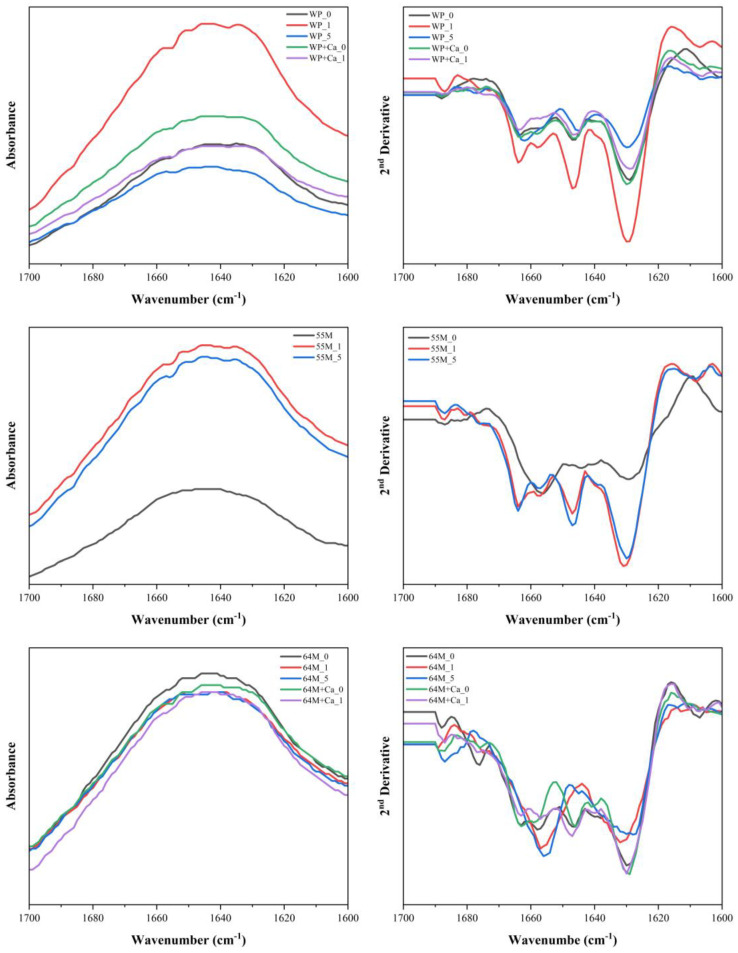
FTIR spectra in the range of 1700–1500cm^−1^ (**left**) and second derivative spectra of the studied samples (**right**). Samples include WP, 55M, and 64M with variations of calcium addition and ultrasound treatment durations (0, 1, and 5 min).

**Figure 5:**
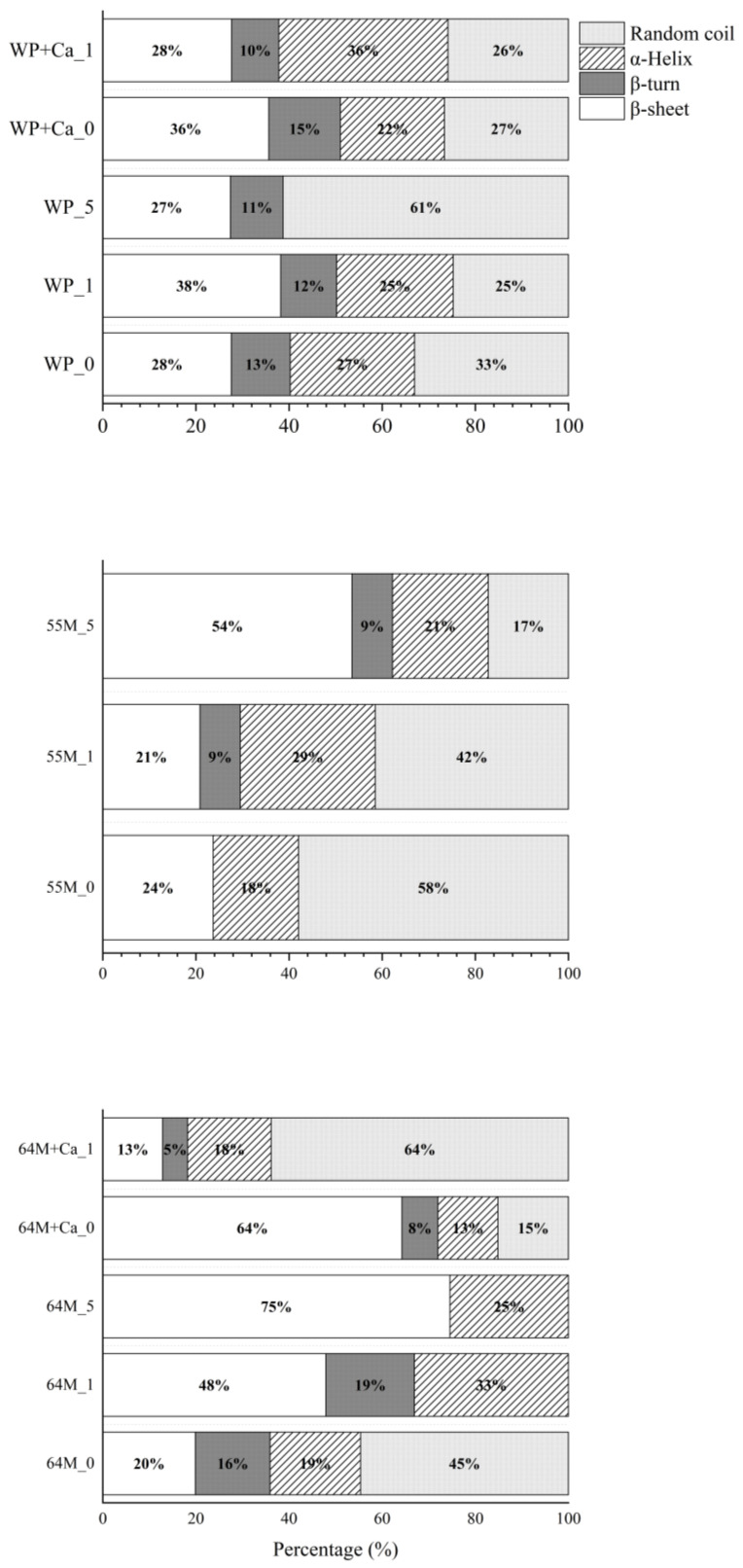
Secondary structure composition of protein in studied samples as determined by FTIR analysis. Samples include WP, 55M, and 64M with variations of calcium addition and ultrasound treatment durations (0, 1, and 5 min).

This region primarily corresponds to the amide II bands, which are indicative of protein backbone conformations [39,56,67]. The second derivative FTIR spectra and secondary structure analysis of the studied samples are shown in Figure 4 and Figure 5. A comparison between the three ratios indicates that 55M experiences more significant structural alterations upon sonication than WP and 64M. The WP sample initially displays peaks at 1678, 1664, 1647, and 1629 cm^−1^ in the 0 min of sonication, indicating a mix of β-sheet and α-helix structures. As sonication progresses to 1 and 5 min, these peaks shift, and new peaks emerge, reflecting structural modifications such as unfolding and aggregation. Specifically, sonication for 1-min results in peaks at 1684, 1647, and 1630 cm^−1^, and at 5 min, peaks at 1683, 1645, and 1630 cm^−1^, indicating an increase in random coil and β-turn content and a decrease in β-sheet structure. The secondary structural analysis confirms these changes, showing a reduction in β-sheet content from 33% at 0 min to 25% and 61% at 1 and 5 min, respectively, with corresponding increases in random coil and β-turn structures. In contrast, the 55M sample, with an equal ratio of whey protein isolate to casein, shows initial peaks at 1674, 1656, 1641, 1632, and 1610 cm^−1^. After 1 min of sonication, these peaks shift significantly. After 5 min of sonication, notable peaks appear at 1664, 1647, and 1631 cm^−1^, suggesting extensive protein denaturation. The secondary structural analysis reveals a pronounced reduction in β-sheet content from 58% at 0 min to 42% and 17% at 1 and 5 min, respectively, and a substantial increase in random coil and β-turn structures, suggesting significant protein denaturation and loss of native conformation compared to WP, consistent with previous studies indicating that ultrasound disrupts ordered β-sheet and enhanced protein unfolding [27,40,68]. This indicates that the 55M sample is more susceptible to structural changes induced by sonication, leading to greater disruption of the native protein structure. The 64M sample, with a higher casein content, initially shows peaks at 1690, 1658, 1646, 1630, and 1616 cm^−1^, indicating a mix of β-sheet and α-helix structures. Sonication induces shifts and the appearance of new peaks, similar to the other samples, but the extent of structural disruption is intermediate between WP and 55M. After 1 min of sonication, peaks appear at 1684, 1657, 1632, and 1609 cm^−1^, indicating partial unfolding and structural modifications. Prolonged sonication for 5 min results in peaks at 1678, 1656, 1628, and 1611 cm^−1^, indicating increased amorphization while retaining more native structure than the 55M sample. The secondary structural analysis shows β-sheet content decreasing from 45% at 0 min to 38% and 25% at 1 and 5 min, respectively, with increases in random coil and β-turn structures. Ultrasound significantly affects the secondary structure of these protein samples, with a clear trend of increasing random coil and β-turn content and decreasing β-sheet content as sonication time progresses, particularly in samples with higher casein content.

When calcium is added to the WP sample, the second derivative spectra display peaks at 1663, 1646, 1630, and 1616 cm^−1^. This suggests a slightly different initial protein structure compared to WP_0, with calcium addition leading to changes in the secondary structure. After 1 min of sonication (WP+Ca_1), the peaks shift to 1664, 1647, and 1629 cm^−1^, indicating structural modifications. The secondary structural analysis shows a decrease in β-sheet content from 27% in WP+Ca_0 to 26% in WP+Ca_1, and an increase in random coil content, suggesting that the combination of calcium and sonication leads to more pronounced structural changes in the proteins. These structural changes are reflected in the increased random coil and β-turn content and decreased β-sheet content with sonication. In the 64M samples, the addition of calcium leads to notable shifts in peaks, with the second derivative spectra showing peaks at 1663, 1646, 1629, and 1616 cm^−1^. These shifts suggest an initial disruption in the protein structure due to calcium addition, causing an increase in random coil content at the expense of β-sheets. The secondary structural analysis confirms this, showing a decrease in β-sheet content from 45% in 64M_0 to 15% in 64M+Ca_0 and an increase in random coil content to 64%. Sonication further amplifies these structural changes. These findings align with previous research suggesting that calcium interacts with casein micelles, altering their structural stability and promoting a more disordered conformation [27]. After 1 min of sonication (64M+Ca_1), the second derivative spectra reveal peaks at 1690, 1657, 1647, 1630, and 1616 cm^−1^, indicating further structural modifications. The secondary structure analysis shows a reduction in random coil content to 18% and an increase in β-sheet content to 64%, suggesting a partial reformation of the β-sheet structure. These findings suggest that calcium addition and sonication induce substantial changes in the secondary structure of proteins, leading to increased β-sheet content and reduced random coil content, similar to the changes observed in WP samples.

## 4. Conclusions

Overall, this study demonstrates that sonication and calcium addition significantly impact on the structural and functional properties of casein-whey mixtures, especially in the presence of lactose. Short-term sonication can enhance solubility by improving particle dispersion, while extended sonication may lead to particle fragmentation and reduced solubility. Calcium stabilizes particle size in casein-rich (64M) systems but promotes aggregation in the whey control formulations. Sonication alters protein secondary structure, increasing β-turn content in WP samples, random coils in 55M, and β-sheets in 64M. Calcium further modulates these structural changes, acting as a cross-linking agent that stabilizes the protein–lactose matrix, as evidenced by increased glass transition temperatures (Tg) and enhanced thermal stability. This effect is particularly pronounced in casein-rich mixtures, where calcium reinforces structural integrity, minimizing changes in particle size and solubility. FTIR analysis confirms that calcium preserves lactose crystallinity, whereas sonication alone promotes amorphization.

These findings have important implications for the dairy industry, particularly in the formulation of dairy powders used in infant formulas, sports nutrition, and functional foods. These findings have important implications for the dairy industry, particularly in the formulation of dairy powders used in infant formulas, sports nutrition, and functional foods. Understanding how calcium mitigates sonication-induced structural disruption in milk proteins–lactose systems provides new strategies for improving shelf life, rehydration properties, and structural stability in high-protein dairy formulations. However, certain limitations must be acknowledged. This study was conducted under specific casein-to-whey ratios and sonication conditions, which may not fully capture the complexity of industrial dairy formulations. Additionally, factors such as pH, ionic strength, and drying conditions may further influence these interactions and should be considered in future research.

## Figures and Tables

**Table 1:** Summary of statistical analysis results for physico-chemical properties of the studied powders.

Responses	Main Effects			Interaction Effects	
	Milk Mixture (A)	Calcium Level (B)	Ultrasound Duration (C)	B × C	A × C
D[3,2]	**	ns	ns	**	***
D[4,3]	ns	ns	ns	ns	ns
d(50)	***	ns	ns	ns	ns
Solubility	ns	ns	ns	ns	ns
Moisture content	ns	***	**	ns	ns
Water activity	ns	**	ns	ns	ns
Bulk density	***	ns	ns	***	ns
Tapped density	***	ns	ns	***	ns
L*	***	*	ns	ns	***
a*	***	***	**	ns	**
b*	***	ns	ns	*	***

Note: The level of statistical significance is presented as *, ** and *** when *p* < 0.05, *p* < 0.01 and *p* < 0.001. ns indicates not significant.

**Table 2:** Color parameters, bulk, and tapped densities for all studied samples.

Samples	Color			Densities		Moisture Content	Water Activity
	L*	a*	b*	Bulk (g/cm^3^)	Tapped (g/cm^3^)	%	
WP_0 min	97.91 ± 0.05 ^AB^ *	−0.47 ± 0.00 ^B^	3.93 ± 0.00 ^F^	0.27 ± 0.02 ^EF^	0.38 ± 0.03 ^D^	3.40 ± 0.29 ^B^	0.27 ± 0.07 ^A^
WP_1 min	97.33 ± 0.13 ^BC^	−0.46 ± 0.02 ^B^	3.80 ± 0.13 ^F^	0.26 ± 0.03 ^F^	0.35 ± 0.01 ^D^	3.30 ± 0.13 ^B^	0.23 ± 0.01 ^A^
WP_5 min	97.48 ± 0.00 ^B^	−0.39 ± 0.01 ^A^	3.80 ± 0.00 ^F^	0.28 ± 0.01 ^DE^	0.38 ± 0.01 ^D^	3.21 ± 0.06 ^B^	0.28 ± 0.05 ^A^
55M_0 min	94.04 ± 0.02 ^E^	−0.80 ± 0.01 ^E^	4.82 ± 0.00 ^B^	0.29 ± 0.02 ^CD^	0.44 ± 0.03 ^C^	3.54 ± 0.19 ^B^	0.22 ± 0.01 ^A^
55M_1 min	94.23 ± 0.02 ^E^	−0.71 ± 0.01 ^D^	5.14 ± 0.01 ^A^	0.32 ± 0.02 ^AB^	0.48 ± 0.01 ^AB^	3.59 ± 0.12 ^B^	0.21 ± 0.01 ^A^
55M_5 min	96.48 ± 0.02 ^D^	−0.88 ± 0.01 ^FG^	4.39 ± 0.00 ^D^	0.29 ± 0.04 ^D^	0.48 ± 0.02 ^AB^	3.28 ± 0.02 ^B^	0.22 ± 0.01 ^A^
64M_0 min	98.23 ± 0.01 ^A^	−0.68 ± 0.01 ^D^	3.82 ± 0.00 ^F^	0.31 ± 0.02 ^BC^	0.49 ± 0.02 ^A^	3.56 ± 0.08 ^B^	0.22 ± 0.01 ^A^
64M_1 min	97.25 ± 0.04 ^BC^	−0.61 ± 0.01 ^C^	4.21 ± 0.02 ^E^	0.33 ± 0.01 ^A^	0.45 ± 0.03 ^BC^	3.21 ± 0.08 ^B^	0.22 ± 0.01 ^A^
64M_5 min	96.64 ± 0.21 ^CD^	−0.84 ± 0.03 ^EF^	5.21 ± 0.01 ^A^	0.33 ± 0.02 ^A^	0.46 ± 0.02 ^ABC^	3.20 ± 0.12 ^B^	0.21 ± 0.01 ^A^
WP+30 mM Calcium_0 min	97.49 ± 0.67 ^B^	−0.58 ± 0.01 ^C^	3.87 ± 0.08 ^F^	0.26 ± 0.02 ^F^	0.38 ± 0.02 ^D^	4.39 ± 0.18 ^A^	0.27 ± 0.01 ^A^
WP+30 mM Calcium_1 min	96.33 ± 0.03 ^D^	−0.45 ± 0.01 ^B^	3.05 ± 0.03 ^G^	0.25 ± 0.04 ^F^	0.35 ± 0.03 ^D^	4.44 ± 0.18 ^A^	0.26 ± 0.01 ^A^
64M+30 mM_0 min	97.23 ± 0.04 ^BC^	−0.83 ± 0.01 ^EF^	4.23 ± 0.03 ^DE^	0.32 ± 0.05 ^AB^	0.44 ± 0.02 ^BC^	4.72 ± 0.41 ^A^	0.28 ± 0.01 ^A^
64M+30 mM_1 min	97.60 ± 0.00 ^AB^	−0.91 ± 0.01 ^G^	4.57 ± 0.00 ^C^	0.25 ± 0.02 ^F^	0.36 ± 0.01 ^D^	4.77 ± 0.16 ^A^	0.26 ± 0.01 ^A^

* Means that do not share letter(s) in the same column are statistically significantly different.

**Figure A1:**
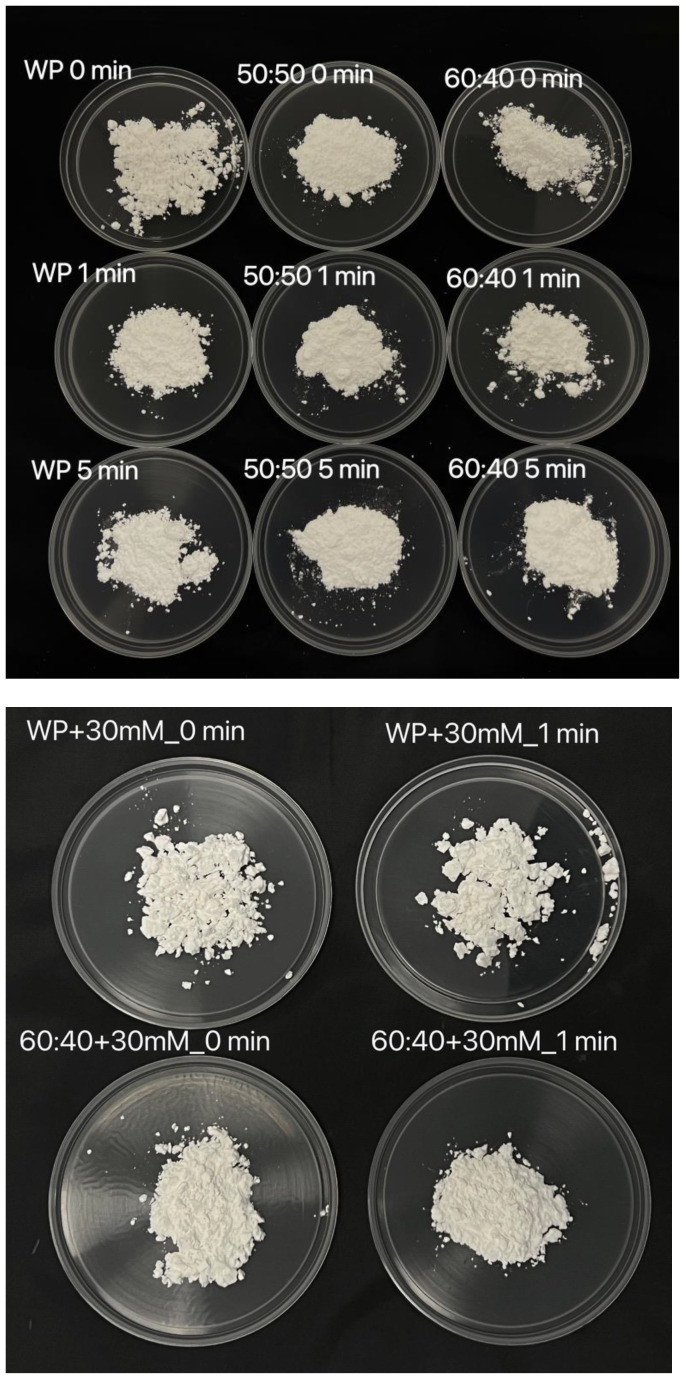
Visual appearances of all studied milk powder samples.

## Data Availability

The original contributions presented in this study are included in the article. Further inquiries can be directed to the corresponding author.

## Appendix B

**Table A1:** Particle size values of all studied milk powder samples.

Samples	Particle Sizes					
	D[3,2], μm	D[4,3], μm	d(10), μm	d(50), μm	d(90), μm	Span
WP_0 min	5.99	22.97	2.95	5.99	22.97	2.04
WP_1 min	6.42	25.20	3.16	9.74	23.00	2.04
WP_5 min	5.20	8.84	2.64	7.51	16.97	1.91
55M_0 min	6.47	11.10	3.25	9.64	20.97	1.84
55M_1 min	6.73	24.40	3.40	10.04	22.93	1.94
55M_5 min	6.40	12.17	3.18	9.74	22.80	2.02
64M_0 min	5.92	10.20	2.99	8.74	19.53	1.89
64M_1 min	6.45	10.77	3.38	9.57	20.03	1.74
64M_5 min	6.64	11.70	3.35	9.98	22.40	1.90
WP+30 mM Calcium_0 min	5.55	10.27	2.79	8.00	19.60	2.10
WP+30 mM Calcium_1 min	6.12	29.60	3.01	8.56	22.03	2.22
64M+30 mM_0 min	6.37	11.13	3.15	9.50	21.40	1.92
64M+30 mM_1 min	6.68	11.87	3.23	10.10	23.07	1.06

## References

[1] Burgain J., Scher J., Petit J., Francius G., Gaiani C. (2016). Links between particle surface hardening and rehydration impairment during micellar casein powder storage. Food Hydrocoll..

[2] Haque M.K., Roos Y.H. (2004). Water plasticization and crystallization of lactose in spray-dried lactose/protein mixtures. J. Food Sci..

[3] Andiç S., Boran G. (2015). Milk Proteins: Functionality and Use in Food Industry.

[4] Balivo A., d’Errico G., Genovese A. (2024). Sensory properties of foods functionalised with milk proteins. Food Hydrocoll..

[5] Deeth H.C., Bansal N. (2018). Whey Proteins: From Milk to Medicine.

[6] McSweeney P.L.H., O’Mahony J.A., Kelly A.L. (2022). Production and Uses of Lactose.

[7] Omoarukhe E.D., On-Nom N., Grandison A.S., Lewis M.J. (2010). Effects of different calcium salts on properties of milk related to heat stability. Int. J. Dairy Technol..

[8] On-Nom N., Grandison A.S., Lewis M.J. (2012). Heat stability of milk supplemented with calcium chloride. J. Dairy Sci..

[9] Pandalaneni K., Amamcharla J.K., Marella C., Metzger L.E. (2018). Influence of milk protein concentrates with modified calcium content on enteral dairy beverage formulations: Physicochemical properties. J. Dairy Sci..

[10] Williams R.P.W., D’Ath L., Augustin M.A. (2005). Production of calcium-fortified milk powders using soluble calcium salts. Lait.

[11] Barone G., Moloney C., O’Regan J., Kelly A.L., O’Mahony J.A. (2020). Influence of calcium fortification on physicochemical properties of whey protein concentrate solutions enriched in α-lactalbumin. Food Chem..

[12] Vyas H.K., Tong P.S. (2004). Impact of Source and Level of Calcium Fortification on the Heat Stability of Reconstituted Skim Milk Powder. J. Dairy Sci..

[13] Wang W., Tan K.W.J., Chiang P.L., Wong W.X., Chen W., Lin Q. (2023). Impact of Incorporating Free Calcium and Magnesium on the Heat Stability of a Dairy- and Soy-Protein-Containing Model Emulsion. Polymers.

[14] Yüksel Z., Erdem Y.K. (2005). The influence of main milk components on the hydrophobic interactions of milk protein system in the course of heat treatment. J. Food Eng..

[15] Singh J., Dean A., Prakash S., Bhandari B., Bansal N. (2021). Ultra high temperature stability of milk protein concentrate: Effect of mineral salts addition. J. Food Eng..

[16] Dissanayake M., Kasapis S., George P., Adhikari B., Palmer M., Meurer B. (2013). Hydrostatic pressure effects on the structural properties of condensed whey protein/lactose systems. Food Hydrocoll..

[17] Haque M.A., Chen J., Aldred P., Adhikari B. (2015). Drying and denaturation characteristics of whey protein isolate in the presence of lactose and trehalose. Food Chem..

[18] Hajihashemi Z., Nasirpour A., Scher J., Desobry S. (2014). Interactions among lactose, β-lactoglobulin and starch in co-lyophilized mixtures as determined by Fourier Transform Infrared Spectroscopy. J. Food Sci. Technol..

[19] Mensink M.A., Frijlink H.W., van der Voort Maarschalk K., Hinrichs W.L.J. (2017). How sugars protect proteins in the solid state and during drying (review): Mechanisms of stabilization in relation to stress conditions. Eur. J. Pharm. Biopharm..

[20] Thomas M.E.C., Scher J., Desobry S. (2004). Lactose/β-Lactoglobulin Interaction During Storage of Model Whey Powders. J. Dairy Sci..

[21] Asaduzzaman M., Mahomud M.S., Haque M.E. (2021). Heat-Induced Interaction of Milk Proteins: Impact on Yoghurt Structure. Int. J. Food Sci..

[22] Mahomud M.S., Katsuno N., Zhang L., Nishizu T. (2017). Physical, rheological, and microstructural properties of whey protein enriched yogurt influenced by heating the milk at different pH values. J. Food Process. Preserv..

[23] Warncke M., Kieferle I., Nguyen T.M., Kulozik U. (2022). Impact of heat treatment, casein/whey protein ratio and protein concentration on rheological properties of milk protein concentrates used for cheese production. J. Food Eng..

[24] Elmonsef Omar A.M., Roos Y.H. (2007). Glass transition and crystallization behaviour of freeze-dried lactose–salt mixtures. LWT Food Sci. Technol..

[25] Li M., Shen M., Lu J., Yang J., Huang Y., Liu L., Fan H., Xie J., Xie M. (2022). Maillard reaction harmful products in dairy products: Formation, occurrence, analysis, and mitigation strategies. Food Res. Int..

[26] Huang Z., Li K., Ma L., Chen F., Hu X., Miao S., Ji J. (2023). The effect of Maillard reaction on the lactose crystallization and flavor release in lactose/WPI/inulin encapsulation. Food Chem. X.

[27] Zhao Y., Saxena J., Cherian V., Silva M., Truong T., Chandrapala J. (2024). Effect of low frequency ultrasound on lactose-protein interactions in protein solution containing different casein to whey protein ratios. Int. J. Food Sci. Technol..

[28] Li K., Wang J., Zhao P., Julian McClements D., Liu X., Liu F. (2024). Effect of ultrasound-assisted Maillard reaction on glycosylation of goat whey protein: Structure and functional properties. Food Chem..

[29] Liu J., Song G., Zhou L., Yuan Y., Wang D., Yuan T., Li L., Yuan H., Xiao G., Gong J. (2023). Recent advances in the effect of ultrasound on the binding of protein−polyphenol complexes in foodstuff. Food Front..

[30] Singh P.K., Huppertz T. (2019). Effect of Nonthermal Processing on Milk Protein Interactions and Functionality.

[31] Shanmugam A., Chandrapala J., Ashokkumar M. (2012). The effect of ultrasound on the physical and functional properties of skim milk. Innov. Food Sci. Emerg. Technol..

[32] Ruecroft G., Hipkiss D., Ly T., Maxted N., Cains P.W. (2005). Sonocrystallization:  The Use of Ultrasound for Improved Industrial Crystallization. Org. Process Res. Dev..

[33] Dincer T.D., Zisu B., Vallet C.G.M.R., Jayasena V., Palmer M., Weeks M. (2014). Sonocrystallisation of lactose in an aqueous system. Int. Dairy J..

[34] Masum A.K.M., Chandrapala J., Huppertz T., Adhikari B., Zisu B. (2020). Influence of drying temperatures and storage parameters on the physicochemical properties of spray-dried infant milk formula powders. Int. Dairy J..

[35] Murphy E.G., Roos Y.H., Hogan S.A., Maher P.G., Flynn C.G., Fenelon M.A. (2015). Physical stability of infant milk formula made with selectively hydrolysed whey proteins. Int. Dairy J..

[36] Anema S.G., Pinder D.N., Hunter R.J., Hemar Y. (2006). Effects of storage temperature on the solubility of milk protein concentrate (MPC85). Food Hydrocoll..

[37] Havea P. (2006). Protein interactions in milk protein concentrate powders. Int. Dairy J..

[38] Markoska T., Huppertz T., Grewal M.K., Vasiljevic T. (2019). FTIR analysis of physiochemical changes in raw skim milk upon concentration. LWT.

[39] Ye M.P., Zhou R., Shi Y.R., Chen H.C., Du Y. (2017). Effects of heating on the secondary structure of proteins in milk powders using mid-infrared spectroscopy. J. Dairy Sci..

[40] Silva M., Zisu B., Chandrapala J. (2018). Influence of low-frequency ultrasound on the physico-chemical and structural characteristics of milk systems with varying casein to whey protein ratios. Ultrason. Sonochem..

[41] Sert D., Mercan E., Dinkul M., Aydemir S. (2021). Processing of skim milk powder made using sonicated milk concentrates: A study of physicochemical, functional, powder flow and microbiological characteristics. Int. Dairy J..

[42] Vercet A., Oria R., Marquina P., Crelier S., Lopez-Buesa P. (2002). Rheological Properties of Yoghurt Made with Milk Submitted to Manothermosonication. J. Agric. Food Chem..

[43] Chever S., Méjean S., Dolivet A., Mei F., Den Boer C.M., Le Barzic G., Jeantet R., Schuck P. (2017). Agglomeration during spray drying: Physical and rehydration properties of whole milk/sugar mixture powders. LWT Food Sci. Technol..

[44] Barkouti A., Turchiuli C., Carcel J.A., Dumoulin E. (2013). Milk powder agglomerate growth and properties in fluidized bed agglomeration. Dairy Sci. Technol..

[45] Shen X., Shao S., Guo M. (2017). Ultrasound-induced changes in physical and functional properties of whey proteins. Int. J. Food Sci. Technol..

[46] Krešić G., Lelas V., Jambrak A.R., Herceg Z., Brnčić S.R. (2008). Influence of novel food processing technologies on the rheological and thermophysical properties of whey proteins. J. Food Eng..

[47] Frydenberg R.P., Hammershøj M., Andersen U., Greve M.T., Wiking L. (2016). Protein denaturation of whey protein isolates (WPIs) induced by high intensity ultrasound during heat gelation. Food Chem..

[48] Chandrapala J., Martin G.J.O., Zisu B., Kentish S.E., Ashokkumar M. (2012). The effect of ultrasound on casein micelle integrity. J. Dairy Sci..

[49] Stanic-Vucinic D., Prodic I., Apostolovic D., Nikolic M., Cirkovic Velickovic T. (2013). Structure and antioxidant activity of β-lactoglobulin-glycoconjugates obtained by high-intensity-ultrasound-induced Maillard reaction in aqueous model systems under neutral conditions. Food Chem..

[50] Cardoso H.B., Wierenga P.A., Gruppen H., Schols H.A. (2018). Maillard induced glycation behaviour of individual milk proteins. Food Chem..

[51] Pugliese A., Cabassi G., Chiavaro E., Paciulli M., Carini E., Mucchetti G. (2017). Physical characterization of whole and skim dried milk powders. J. Food Sci. Technol..

[52] Semo E., Kesselman E., Danino D., Livney Y.D. (2007). Casein micelle as a natural nano-capsular vehicle for nutraceuticals. Food Hydrocoll..

[53] Jouppila K., Roos Y.H. (1994). Glass Transitions and Crystallization in Milk Powders. J. Dairy Sci..

[54] Zhou P., Labuza T.P. (2007). Effect of Water Content on Glass Transition and Protein Aggregation of Whey Protein Powders During Short-Term Storage. Food Biophys..

[55] Sánchez-García Y.I., García-Vega K.S., Leal-Ramos M.Y., Salmeron I., Gutiérrez-Méndez N. (2018). Ultrasound-assisted crystallization of lactose in the presence of whey proteins and κ-carrageenan. Ultrason. Sonochem..

[56] Ostrowska-Ligęza E., Górska A., Wirkowska M., Koczoń P. (2012). An assessment of various powdered baby formulas by conventional methods (DSC) or FT-IR spectroscopy. J. Therm. Anal. Calorim..

[57] Pugliese A., Paciulli M., Chiavaro E., Mucchetti G. (2019). Application of differential scanning calorimetry to freeze-dried milk and milk fractions. J. Therm. Anal. Calorim..

[58] Szulc K., Nazarko J., Ostrowska-Ligęza E., Lenart A. (2016). Effect of fat replacement on flow and thermal properties of dairy powders. LWT Food Sci. Technol..

[59] Zhang H., Takenaka M., Isobe S. (2004). DSC and electrophoretic studies on soymilk protein denaturation. J. Therm. Anal. Calorim..

[60] Listiohadi Y., Hourigan J.A., Sleigh R.W., Steele R.J. (2009). Thermal analysis of amorphous lactose and α-lactose monohydrate. Dairy Sci. Technol..

[61] Della Bella A., Müller M., Soldati L., Elviri L., Bettini R. (2016). Quantitative determination of micronization-induced changes in the solid state of lactose. Int. J. Pharm..

[62] Badal Tejedor M., Pazesh S., Nordgren N., Schuleit M., Rutland M.W., Alderborn G., Millqvist-Fureby A. (2018). Milling induced amorphisation and recrystallization of α-lactose monohydrate. Int. J. Pharm..

[63] Corrigan D.O., Healy A.M., Corrigan O.I. (2002). The effect of spray drying solutions of polyethylene glycol (PEG) and lactose/PEG on their physicochemical properties. Int. J. Pharm..

[64] Wijayasinghe R., Vasiljevic T., Chandrapala J. (2023). Unraveling the Influences of Sodium, Potassium, Magnesium, and Calcium on the Crystallization Behavior of Lactose. Foods.

[65] Wu L., Miao X., Shan Z., Huang Y., Li L., Pan X., Yao Q., Li G., Wu C. (2014). Studies on the spray dried lactose as carrier for dry powder inhalation. Asian J. Pharm. Sci..

[66] Kougoulos E., Marziano I., Miller P.R. (2010). Lactose particle engineering: Influence of ultrasound and anti-solvent on crystal habit and particle size. J. Cryst. Growth.

[67] Kher A., Udabage P., McKinnon I., McNaughton D., Augustin M.A. (2007). FTIR investigation of spray-dried milk protein concentrate powders. Vib. Spectrosc..

[68] Liyanaarachchi W.S., Ramchandran L., Vasiljevic T. (2015). Controlling heat induced aggregation of whey proteins by casein inclusion in concentrated protein dispersions. Int. Dairy J..

